# Knowledge translation research in population health: establishing a collaborative research agenda

**DOI:** 10.1186/1478-4505-7-28

**Published:** 2009-12-10

**Authors:** Christian Dagenais, Valéry Ridde, Marie-Claire Laurendeau, Karine Souffez

**Affiliations:** 1 Université de Montréal, 555 René-Lévesque Ouest, Montreal, Quebec, Canada; 2 Université de Montréal, Montreal, Quebec, Canada; 3 Institut national de santé publique du Québec, Montreal, Quebec, Canada

## Abstract

**Background:**

Despite the increasing mobilization of researchers and funding organizations around knowledge translation (KT) in Canada and elsewhere, many questions have been only partially answered, particularly in the field of population health. This article presents the results of a systematic process to draw out possible avenues of collaboration for researchers, practitioners and decision-makers who work in the area of KT. The main objective was to establish a research agenda on knowledge translation in population health.

**Methods:**

Using the Concept Mapping approach, the research team wanted to identify priority themes for the development of research on KT in population health. Mapping is based on multivariate statistical analyses (multidimensional scaling and hierarchical cluster analysis) in which statements produced during a brainstorming session are grouped in weighted clusters. The final maps are a visual representation of the priority themes of research on KT. Especially designed for facilitating consensus in the understanding and organization of various concepts, the Concept Mapping method proved suitable for achieving this objective.

**Results:**

The maps were produced by 19 participants from university settings, and from institutions within the health and social services network. Three main perspectives emerge from this operation: (1) The evaluation of the effectiveness of KT efforts is one of the main research priorities; (2) The importance of taking into consideration user contexts in any KT effort; (3) The challenges related to sharing power for decision-making and action-taking among various stakeholder groups. These perspectives open up avenues of collaboration for stakeholders who are involved in research on KT. Besides these three main perspectives, the concept maps reveal three other trends which should be emphasized.

**Conclusion:**

The Concept Mapping process reported in this article aimed to provoke collective reflection on the research questions that should be studied, in order to foster coherence in research activities in the field of population health. Based on this, it is appropriate to continue to support the development of research projects in KT and the formation of research teams in this field. Research on KT must lead to concrete outcomes within communities that are interested in the question.

## Background

Decisions and judgements concerning social problems are becoming increasingly evidence-based [1]. This development has manifested itself in the emergence in recent decades of numerous new approaches, including knowledge transfer, knowledge translation and knowledge mobilization, among others. In a study of 33 funding organizations in 9 countries, Graham and his colleagues identified 29 different terms used to refer to the concept of "knowledge to action" [2]. The authors point out that these terms are often used interchangeably, and that a given term may have different meanings for different organizations. The Canadian Institutes for Health Research (CIHR) define knowledge translation as: "the exchange, synthesis and ethically-sound application of knowledge - within a complex system of interactions among researchers and users - to accelerate the capture of the benefits of research for Canadians through improved health, more effective services and products, and a strengthened health care system" [3].

Processes and strategies that lead to the use of evidence based knowledge are multiple and varied [1]. Graham and his colleagues [2] propose to separate these mechanisms into two cycles. The first cycle focuses on knowledge production that could be used by different practitioners and include systematic reviews as well as tools and products such as practice guides, and tools to help with decision making. The second cycle centers on actions necessary for the application of knowledge produced. Work from these authors helped identify over 60 models or theoretical frameworks to support knowledge translation. These models or theories present eight common characteristics: 1) identification of a problem or a need, 2) identification of pertinent knowledge to resolve a problem or answer a need, 3) adaptation of knowledge to local context, 4) examination of barriers to use, 5) choice and implementation of an intervention which promotes use, 6) follow-up of use, 7) evaluation of effects or impacts of use, and 8) activities to support and maintain use.

Being multi-disciplinary in nature, the research on knowledge translation (KT) seeks to better understand the dynamic that fosters the use of research and its impact on public policies, decision making, and professional practice [1]. The motivation for the CIHR to finance the development of information about KT stems from a movement initiated by the Canadian Health Services Research Foundation (CHSRF). In the last several years the CHSRF and their experts developed a large body of theoretical and empirical work on KT, provided financing for experiments on the subject, and organised training for health system managers [4,5].

Despite the increasing mobilization of researchers and funding organizations around knowledge translation (KT) in Canada and elsewhere, some questions have been only partially answered, particularly in the field of population health. Firstly, (1) there is still very little evidence-based data on the effectiveness of KT in changing practices and decision-making processes, and ultimately, in improving population health and well-being [1]. Empirical research on the effects of KT efforts focuses primarily on direct effects such as changes in practices [6,7], the improvement of skills [8] and changes in the attitudes of users [7-9]. However, very few studies report results on the effects on populations targeted by interventions [9]. As well, evidence for the effects of KT in the social field often remains compartmentalized by discipline [10-12]. Secondly, (2) it is possible to identify a multitude of conditions that foster KT. These conditions can be grouped into 4 categories: 1) **Individuals**: the perception among potential users that the proposed change is worthwhile [13], congruence between the needs, values and beliefs of users, and characteristics of the proposed change [14-17]; 2) **Organizations**: support for the implementation of the change [18,19], leadership on the part of key stakeholders [17,19,20], and the culture of the setting [17,19-23]; 3) **Strategies **to increase uptake: the involvement of potential users in research activities [24,25], and the development of explicit recommendations for action [26-28]; 4) **Resources available**: necessary time and materials [20], and human and financial resources [29]. But these conditions vary considerably depending on the type of knowledge being transferred and on the context of usage [27]. Lastly, (3) there is very little evidence on what strategies are effective for different types of users and different types of use [1,30-32]. In their overview of the literature on this topic, Grimshaw and his colleagues [33] conclude that no strategy is effective under all circumstances. This conclusion is consistent with that of Nutley, Walter & Davies [34] who, in a cross-sector literature review, compare three models of evidence-based practice: the research-based practitioner model, the embedded research model, and the organizational excellence model. The authors conclude that "the ideas contained within each of these models are likely to be appropriate at different times and for different service settings." (p. 552).

Throughout the academic world, research on knowledge translation is emerging as a distinct field. Although KT research is a rapidly growing field of study, it is also a scattered one, characterized by a wide variety of research interests [2]. In order to identify the priority themes of research on KT, the Quebec Population Health Research Network (QPHRN) - a network of population health researchers based in the province of Québec (Canada) - initiated a series of consultations at the beginning of 2007.

Population health is part of an approach that studies the determinants of health (e.g., income and social environment) and strives for a better understanding of the mechanisms through which inequalities occur among different population groups, with a view to improving overall population health [35,36].

The population health field is vast, complex and interdisciplinary [37]. The social problems addressed are complex and often difficult to approach through an experimental research protocol, making it difficult to produce evidence, as it is understood by clinical practice. Accordingly, KT strategies implemented in this field must take into account the type of evidence used and the context within which it is applied. This context differs significantly from that of physicians, who interact with patients on a more individual basis[38].

The objective of the research team was to identify priority themes for the development of research on knowledge translation in population health and to draw out possible avenues of research. The results shed light on the need for fostering better synergy among research efforts on KT. This affirmation was recently endorsed by other researchers in KT who also put forward perspectives for research to develop in this field. For example, Kitson and Bisby [39] identified three types of interests in the research on KT: 1) theoretical (conceptual framework, definitions, etc.); 2) policy (role of funders, transfer of learning, etc.); and 3) methodology (methods, outcome measures, knowledge management, etc.). The process initiated by the QPHRN in 2007 is therefore part of a wider reflection on the evolution of the KT field and on the development of a common research agenda.

## Method

The Concept Mapping (CM) approach was chosen in order to achieve the consultative process led by the KT research team. This method is specially designed for facilitating consensus in the understanding and organization of various concepts [40,41]. Since the procedure has been extensively described in the literature [42-44], this article will only briefly describe the process and then concentrate on the findings.

### Participants

Nineteen specialists were selected based on the major environments in which research on knowledge translation in population health is being carried out in Quebec so as to represent the concerns of these areas vis-ΰ-vis KT (purposeful sampling: [45]). We used purposeful sampling to identify and recruit 19 "specialists". Patton [45] states that "the logic and power of purposeful sampling lies in selecting information-rich cases for study in depth. Information-rich cases are those from which one can learn a great deal about issues of central importance to the purpose of the research..." (p. 169). As a first step, the leading researchers in the province of Quebec working on KT were convened for an informal preliminary meeting, held on February 22, 2006. They had been previously identified by the QPHRN based on their research interests and their work on KT. These individuals represented the main settings in Quebec for research and inquiry into KT in population health: universities, research institutes and centres, health and social services agencies, and public health organizations.

Accordingly, the main criterion for selecting the Concept mapping respondents was that they be representative of their "stakeholder group" and action context. Nine participants came from university settings, and 10 from institutions within the health and social services network. University participants came from a variety of disciplines: psychology, health administration, nursing science, management, medicine, political science, and public and population health. Participants from practice and intervention environments represented the various stakeholders involved in the KT processes. They included two general managers of regional health agencies, three decision-makers responsible for KT in the health and social services system, one at the provincial level, one at the regional, and one at the local level, the CEO of a Federal Collaboration Center on Public Policies specialized in KT, two knowledge brokers in health and social services organizations at the regional and local levels et and one coordinator of a Québec KT Centre in Mental Health. These individuals had all previously participated in studies on KT.

### Procedure

#### The construction of concept maps is carried out in five steps

##### Step 1: Focusing on the question

The concept maps are based on information that is produced to answer a single question. For the purposes of our study, the question, prepared by the research team, was formulated as follows: "What should research in knowledge translation concentrate on in order to make sharing and using knowledge about population health more fruitful?"

##### Step 2: Brainstorming session

To answer this question, the group of specialists was invited to collectively produce a series of statements during a brainstorming session. The usual rules for brainstorming applied, with the moderator encouraging participants to produce as many statements on the question as possible and ensuring that all participants had the opportunity to express themselves [46,47]. The moderator also specified that the value or relevance of statements could not be discussed, but that questions could be asked to clarify their meaning. In total, 104 statements were formulated by the participants during the initial hour and a half of this first meeting.

##### Step 3: Rating and sorting statements

The process continued in the second part of the first meeting with two other tasks that required participants to work individually for about an hour. First, participants were asked to score each of the 104 statements on a scale of 1 to 5, where 5 meant "very important", to rank their relative importance. Next, each participant was instructed to group the statements into categories that were meaningful to them. Each category had to contain statements that participants viewed as priorities for research on KT.

##### Step 4: Data analysis

Three statistical operations are necessary for producing concepts maps. First, multidimensional scaling [48] was used to chart each of the original 104 statements in terms of the correlational distance separating them. This distance is determined by the number of times one statement is associated with another by participants. Next, a hierarchical cluster analysis [49] was conducted to group elements representing similar concepts into clusters. Hierarchical cluster analysis makes it possible to produce any number of clusters, going from 104 (in our study), which would represent each of the formulated statements, to a single cluster, which would group all the statements together. Analysts must determine the most appropriate number of clusters for the second group session, in which participants are asked to interpret the results obtained. The final operation consists of calculating the average score assigned to each statement by participants. These statistical analyses make it possible to produce preliminary maps. In these first analyses, the research team noticed substantial differences in conception between participants from the universities and those from the health and social services network. To explore these differences, the research team produced two preliminary maps and split the group into two in order to analyse the results separately, so that the views from each environment would emerge.

##### Step 5: Interpretation of maps

At the second meeting, the two sub-groups (academic and health and social services groups) were present so as to reach a consensus on the general meaning of the clusters and their naming. The configuration of the final map for each of the sub-groups resulted essentially from the cluster categorization exercise. The importance of each cluster is represented on the map by the number of strata that compose it (from one to five). This importance is determined by the average of the scores given by participants to each of the statements. However, certain clusters contain more dispersed statements than others, and as a result they vary considerably in size. The size of clusters is not proportional to their importance; instead, their importance is represented by the number of strata they comprise.

All 19 participants (academic = 9; health and social services = 10) were present at the first session (brain storming, rating and sorting). However, fewer attended the second session (academic = 5; health and social services = 10).

## Results

### Map for the Academic Group

**Figure 1:**
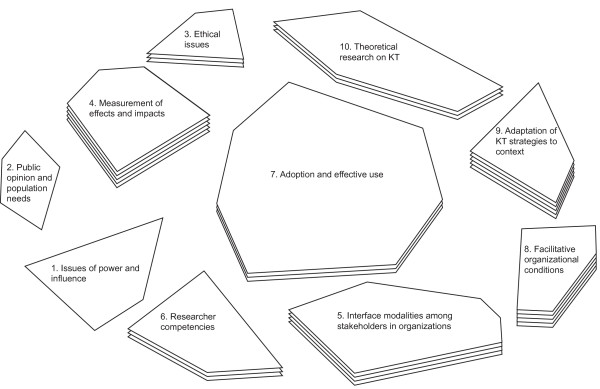
**Final map for the Academic Group**.

The map in figure 1 shows the research priorities identified by participants from academic settings. Table S1, Additional file 1 shows the items in each cluster and the average scores given to each of them.

The four most important research priorities identified are: 1) Facilitative organizational conditions (cluster 8: Institutional barriers and facilitating factors experienced by researchers and users, Capacity of KT mechanisms to adjust to the decision-making window, etc.); 2) Measurement of effects and impacts (cluster 4: Measuring the outcomes and benefits of KT, The impact of KT on population health, etc.); 3) Adaptation of KT strategies to context (cluster 9: The integration of existing evidence and knowledge within organizations, The optimal relationship between tacit and explicit knowledge, etc.); 4) Interface modalities among stakeholders in organizations (cluster 5: The relevance of various KT strategies in relation to the needs of different types of users, The development of leadership roles related to KT on the ground, etc.).

Of these four research priorities, clusters 8, 9 and 5 share certain similarities. All of them pertain to the factors and conditions that foster increased use of research. The map bears witness, moreover, to this relationship, since these three clusters are near each other spatially. The fourth one, which is located to the extreme left of the map, groups the statements that refer to the evaluation of the effectiveness of different strategies for increasing research utilization.

### Map for the Health and Social Services Group

**Figure 2:**
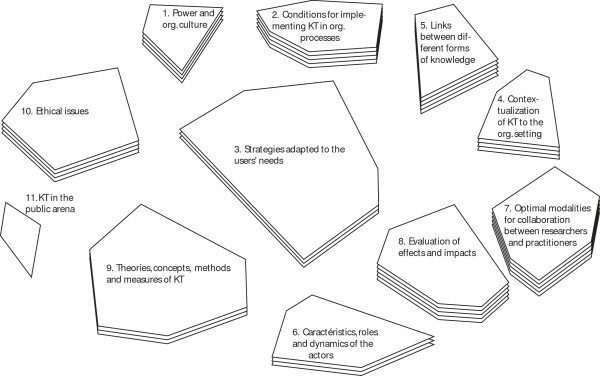
**Final map for the Network Group**.

For their part, participants in the "health and social services" group were mainly concerned with understanding how to integrate new knowledge resulting from research into knowledge that already existed in organizations (figure 2). The emphasis was on relationships among stakeholders who are involved in KT, as well as on the optimal forms that their interactions could take. Table S2, see Additional file 1 shows the items in each cluster and the average scores given to each of them.

In fact, the clusters 5: Links between the different forms of knowledge (The fit between scientific knowledge and field-based knowledge, Issues and differentiated strategies with regards to tacit and explicit knowledge, etc.), 2: Conditions for implementing KT in organizational processes (Strategies that promote continuity in the use of knowledge, The attributes of research-based knowledge that make it shareable and usable, etc.) and 7: Optimal modalities for collaboration between researchers and practitioners (The development of leadership roles related to KT on the ground, The question of relational contexts that foster the use of knowledge, etc.), which represent three of the four most important research priorities identified by the health and social services group of participants, reflect their desire to better determine the ability of various communities to integrate scientific knowledge into their practices. The statements in these clusters demonstrate the predominance of this theme in the health and social services map.

## Discussion

The maps thus obtained are based entirely on the responses and statements of participants, and constitute graphical representations of their conceptions of research priorities in KT. Despite the configurations proper to each, the two maps share several common perspectives on research development.

### Main Research Priorities

Three main perspectives emerge from the examination of the four most important clusters for each of the two maps. They open up avenues of collaboration for stakeholders who are involved, directly or indirectly, in research on KT.

#### Evaluation of the Impacts and Outcomes of KT

Participants in both groups consider evaluation of the effectiveness of KT efforts as one of the main research priorities. They deem it important to develop research on the structural impacts, within health systems, of evidence-based practices. The environment appears favourable for undertaking such studies, since funding organizations are providing increasing support for work on the outcomes of KT, particularly in the area of population health in Canada. Furthermore, given the multitude of new KT strategies and models being implemented to bring research and practitioner communities closer together, organizations now want to know whether their investments are making a difference. They also want to be able to identify strategies to improve program and policy performance [1,50].

#### Contextualization of Knowledge Translation

Concept Mapping results call special attention to the notion of context. Participants from both groups recognized the specific character of KT in terms of communities and organizational cultures. The lack of data on conditions for implementation in organizational processes (map generated by the health and social services group) and on the adaptation of these strategies based on application contexts and facilitating organizational conditions (map generated by the academic group), justifies research efforts on this subject. Studies on the conditions for research utilization show, moreover, the importance of taking into consideration user contexts for action and application [25,33,51-55].

#### Interface Modalities between Stakeholder Groups

This theme is evidence of a growing and generalized need for emphasizing applied research that targets KT for the purpose of backing decision-making and action-taking. Among other things being investigated are optimal KT strategies, new functions to develop in organizations, the strategic positioning of stakeholders within networks, the type of competencies required in professionals, and the preferred platforms for collaboration among researchers, decision makers, and caregivers.

Organizations must deal with new challenges related to sharing power for decision-making and action-taking among various stakeholder groups. The lack of theoretical and empirical knowledge on these questions is obliging KT practitioners to proceed in an exploratory fashion for the time being. In this respect, constructivist theories on social epistemology can be very relevant in reorienting research approaches and questions in KT [56]. They deal specifically with the decision-making influence of producers and users of knowledge, the power relationships between the two, the impacts of various KT strategies on the dynamics of power in organizations, and the very nature of knowledge that can or should be exchanged.

### Emerging Trends

Besides these three main perspectives, the concept maps reveal three other trends which should be emphasized.

Firstly, there was consensus between both groups on the Ethical Issues theme, which received a rather high average score. Participants believe that research can play a useful role in encouraging reflection on ethical issues. KT practices raise numerous questions for stakeholders, especially since they are confronted with complex situations for which there is still no normative framework. There are questions about the respective roles and responsibilities of researchers, knowledge brokers, and decision makers in the decision-making process; the appropriate moment for transferring knowledge; how to monitor KT activities and whether they should even be monitored. These questions all represent topics that KT researchers could explore. Some authors are heralding a "fourth wave" in KT research, which will be characterized, amongst other things, by greater attention to ethical concerns and social justice issues [57].

Secondly, the health and social services group's map contained a cluster on the "needs of the community", which had no counterpart in the map of the academic group. However, congruence between the needs of potential users and the characteristics of change [17,58,59] as well as the congruence between the project for change and the values and beliefs of the potential user [51,58,60,61] appear to be conditions that determine research utilization. For this reason, several authors [62-64] suggest that researchers lean more towards the point of view of users and develop a better understanding of the context that pertains to each group of targeted users.

Thirdly, clusters that group items related to public involvement (Public opinion and needs of the population and KT in the public arena) were considered to be less important by both groups of participants. A possible explanation is that there were no representatives from the public who participated in the exercise, which constitutes one of the limitations of this study. However, Abelson and colleagues [65] show that citizens want increased participation in decision-making in the public arena, and that they are very critical of their participation experiences. They are able to identify the improvements needed in processes for exchanging information between decision makers and citizens; they view themselves on the same level as decision makers in terms of being sources of information, and they want to be consulted. However, the needs of citizens will only be taken into consideration in KT processes to the extent that their access to information and participation in decision-making is facilitated.

Confirmation of the ecological validity of Concept Mapping Results by a Larger Pool of Stakeholders

Ecological validity is defined as 'the extent to which the environment experienced by the participants in a scientific investigation has the properties it is supposed or assumed to have by the investigator' [66]. In other words, the QPHRN research team wanted to be reasonably sure the CM results apply to the 'real world'. As part of a half-day seminar in March 2007, a wider group of thirty key stakeholders from various communities concerned by KT in population health (universities, health and social service centres and agencies, public health departments, research institutes, funding organizations, etc.) was invited to comment on the meaning and interpretation of the concept mapping results.

All participants recognized the relevance of the exercise. The research areas identified were very indicative of the actual concerns of the researchers, decision-makers, and caregivers who are involved in research on KT. According to the participants, the conclusions paint an interesting portrait of the current state of affairs, enriched by the similarities and differences between the academic and the health and social services groups.

According to participants, the issue is no longer the "What?" of KT, but rather the "How?" How is KT happening in organizations? How can research findings be transformed into knowledge that can bring about change? How can appropriate strategies be put into place with regards to the context and conditions of the community in question? KT practitioners are becoming increasingly aware that the integration of scientific knowledge into organizations depends on utilization practices. That is why seminar participants, like the participants in the Concept Mapping exercise, insisted on the need for researching usage contexts and the conditions necessary for implementing research-based knowledge.

### Limitations

While the results produced by the CM process admittedly were relevant to the participants of this study, they presented certain limitations. The sample size was relatively small, since this method is not applicable to larger groups. To compensate for this limitation, results were validated by a group of 30 stakeholders. Being forced to separate the group into two and to produce two separate maps could also be considered a limitation. This decision, although complicating the interpretation of the results, highlighted the differences in conception between these two groups of participants. Overall, although we do not know to what extent the research priorities identified apply to other fields of practice, we consider that this method proved appropriate in the context of the current study.

## Conclusion

The field of KT has experienced enormous growth over the past few years. The number of studies and publications on the subject is steadily increasing. Several teams are working in this area and various research projects are underway with regards to the needs identified by different communities. These efforts, however, remain scattered and few teams have the visibility and support necessary to consolidate and grow.

In this respect, the Concept Mapping process reported in this article aimed to provoke collective reflection on the research questions that should be studied with regards to KT, in order to foster coherence in research and intervention activities in the field of population health. Using a systematic and proven technique to achieve this goal has contributed to:

1. More structuring in the conceptual representation of KT;

2. Providing key knowledge translation stakeholders with the opportunity to situate themselves within the field;

3. Bringing to light concerns shared by stakeholders in the university and socio-health communities by providing them with an opportunity to come together;

4. Increased targeting of research efforts in this domain.

Based on this, it is appropriate to continue to support the development of research projects in KT and the formation of research teams in this field. Research on KT must lead to concrete outcomes within communities that are interested in the question.

To date, a publication reporting on the results of the concept mapping has also been produced and distributed within the Quebec health network and to funding organizations. The concept mapping process was intended as a first step toward providing more structure for research on KT in Quebec. The QPHRN now wishes to promote greater synergy between research efforts on KT around the themes identified. To do so, it has provided funding to support the development of a research team on KT. The proposed research program concerns the evaluation of the impacts and benefits of KT.

The QPHRN's objectives also include the creation of other mechanisms to facilitate meetings and exchange among researchers in the field, with the aim of furthering the theory and practice of KT. A number of initiatives are planned for the coming years, including the formation of issue clusters to address the key themes identified through the concept mapping process, and networking with Canadian, European and international stakeholders to promote the research agenda.

## Competing interests

The authors declare that they have no competing interests.

## Authors' contributions

CD made substantial contributions to the conception and design of the study, the acquisition of the data and the interpretation of the data. He drafted the final manuscript.

VR made substantial contributions to the conception and design of the study and to the interpretation of the data. He was involved in critically revising the manuscript for important intellectual content and gave final approval of the version to be published.

MCL made substantial contributions to the conception and design of the study, the acquisition of the data and the interpretation of data. She was involved in critically revising the manuscript for important intellectual content and gave final approval of the version to be published.

KS made substantial contributions to the conception and design of the study, the acquisition of the data and the interpretation of the data. She was involved in drafting the manuscript and gave final approval of the version to be published.

## Supplementary Material

Additional file 1 Table S1 and Table S2 - The data provided represent the statements by cluster for the academic group and for the health and social services group. Click here for file
