# Supplementary material for: Knowledge translation research in population health: establishing a collaborative research agenda
Source: Health Res Policy Syst. 2009 Dec 10;7:28. doi: 10.1186/1478-4505-7-28 (PMC2796997; doi:10.1186/1478-4505-7-28)
Supplement: Additional file 1 — Table S1 and Table S2 - The data provided represent the statements by cluster for the academic group and for the health and social services group. [file 1478-4505-7-28-S1.DOC]

# Additional file 1

# Tables

## Table S1: Statements by cluster for the Academic Group

|  | **Cluster 1. Issues of power and influence** |  |
| --- | --- | --- |
| # | Statement | Average rating |
| 71 | The use of research findings in relationships of power within organizations | 3.89 |
| 17 | Analysis of power (decision-making, influence, and recommendation) issues between researchers and caregivers | 3.00 |
| 28 | The influence of an increasing demand for KT on the development of scientific careers, the development of methods, and on scientific performance | 2.78 |
| 13 | Monitoring processes for decision-making elements | 2.67 |
| 1 | The study of power sharing in decision making and action taking | 2.56 |
|  | **Average:** | **: 2.98** |
|  | **Cluster 2. Public opinion and population needs** |  |
| 72 | The role of patient populations and communities in KT strategies | 3.89 |
| 100 | The impact of new communication technologies on how the population expresses its needs | 3.33 |
| 65 | The place of formal knowledge in the evolution and moulding of public opinion | 3.11 |
| 30 | The place of population needs in supporting choices | 3.11 |
| 21 | Methodological skills of journalists in understanding science | 2.89 |
| 70 | Analysis of the population’s reception of speeches given by KT leaders (media, health agencies, etc.) | 2.44 |
| 66 | Media discourse on KT research activities | 2.44 |
| 69 | Public opinion of research on KT | 1.89 |
|  | **Average:** | **2.89** |
|  | **Cluster 3. Ethical issues** |  |
| 73 | The ethical challenges related to KT | 3.44 |
| 90 | The ethics of the role of the KT professional within the decision-making process | 3.44 |
| 94 | The responsibility of researchers to share knowledge resulting from their own research | 3.33 |
| 96 | The power held by knowledge brokers and the ethical issues this raises | 2.78 |
|  | **Average:** | **3.25** |
|  | **Cluster 4. Measurement of effects and impacts** |  |
| 76 | Economic impact of KT practice on health systems | 4.22 |
| 98 | Measuring the outcomes and benefits of KT | 3.78 |
| 3 | Assessment of experiments underway in order to identify best practices | 3.67 |
| 89 | The impact of KT on population health | 3.67 |
| 82 | Transfer guides which are not evidence-based | 3.56 |
| 55 | The proportion of resources allocated to KT in relation to the proportion of resources allocated to knowledge production | 3.33 |
| 97 | The impact of new communication technologies on KT | 3.22 |
| 31 | The impact on knowledge producers of participating in KT | 3.22 |

|  | **Average:** | **3.58** |
| --- | --- | --- |
|  | **Cluster 5. Interface modalities among stakeholders in organizations** |  |
| 9 | Modalities of collaboration between researchers and practitioners that foster the development of new practices | 4.11 |
| 34 | The relevance of various KT strategies in relation to the needs of different types of users | 4.11 |
| 7 | Roles and competencies to develop in practitioner settings | 4.00 |
| 5 | The role of users in KT | 3.67 |
| 67 | The fit between the portrayals of knowledge by practitioners and researchers | 3.56 |
| 83 | The characteristics of optimality in interdisciplinary teams engaged in KT | 3.56 |
| 52 | The active role of practitioners and decision-makers in KT strategies | 3.44 |
| 25 | Strategies and processes for networking and partnership that foster the co-generation of knowledge | 3.33 |
| 64 | The role of communities of practice in creating synergy between tacit and explicit knowledge | 3.33 |
| 63 | The professional and relational qualities of KT professionals | 3.22 |
| 41 | The development of leadership roles related to KT on the ground | 3.22 |
| 10 | Receptivity of caregivers to new knowledge that will transform practice | 3.22 |
| 84 | The optimal modalities of KT: the role of researcher versus that of KT professional | 3.22 |
| 14 | Methodological skills of organization-based professionals in identifying relevant research work | 3.11 |
| 51 | Development of a KT culture in order to reduce resistance to change | 2.78 |
| 49 | Optimal positioning of KT professionals within the network | 2.56 |
|  | **Average:** | **3.40** |
|  | **Cluster 6. Researcher competencies** |  |
| 6 | Roles and competencies to develop in research settings | 4.00 |
| 20 | Identify the skills of researchers in translating their data for different settings | 3.89 |
| 39 | The skills to develop in researchers for communicating with journalists and the media | 3.22 |
| 27 | Anticipation of the needs of users so that research findings are available when needed | 3.11 |
| 54 | Skill development in researchers for producing research on KT | 3.00 |
| 32 | The ability to listen to the real and current needs of practitioners and researchers | 2.89 |
|  | **Average: 3.35** |  |
|  | **Cluster 7. Adoption and effective use** |  |
| 15 | Analysis of the degree of adoption of proven practices and their determinants | 4.33 |
| 19 | Transformation of clinical settings to support transformation in practices | 3.89 |
| 101 | The fit between scientific knowledge and field-based knowledge | 3.89 |
| 60 | The concordance between the values stated by institutions and their KT practices | 3.67 |
| 23 | Differentiated evaluation of the knowledge needs of the various stakeholders within the network | 3.56 |
| 58 | Systemic issues surrounding the adoption and use of knowledge | 3.22 |
| 8 | The distinctive place of knowledge in decision-making processes through various organizational factors | 3.22 |
| 61 | The strategic use by decision-makers of research-based knowledge | 3.22 |
| 57 | Study of the values and attitudes of the heads of universities and funding organizations towards KT | 3.11 |
| 93 | The availability of explicit scientific knowledge in organizations | 3.11 |
| 81 | The impact of cultural differences (societal cultures) on KT | 3.00 |
| 43 | Elements of recognition of this new practice among researchers as well as among users and their organizations | 2.89 |
| 24 | The views of researchers and practitioners on the deliberations necessary for using knowledge | 2.78 |
| 35 | The place of KT research in clinical informatics | 2.56 |
|  | **Average:** | **3.32** |
|  | **Cluster 8. Facilitative organizational conditions** |  |
| 4 | The question of relational contexts that foster the use of knowledge | 4.11 |
| 79 | Organizational conditions for implementing KT activities | 3.89 |
| 99 | Strategies that promote continuity in the use of knowledge | 3.89 |
| 37 | The role of various KT strategies in developing competencies in public health in organizations | 3.78 |
| 18 | Institutional barriers and facilitating factors experienced by researchers and users | 3.78 |
| 42 | An understanding of the processes of change in new public health practices | 3.67 |
| 95 | The importance of a relationship of trust between researchers and practitioners in the use of research-based knowledge | 3.67 |
| 12 | Capacity of KT mechanisms to adjust to the decision-making window | 3.67 |
| 26 | Conditions for bringing professional cultures together in the context of establishing networks | 3.44 |
| 2 | Preliminary capacities to build within systems (generation, dissemination, absorption) | 3.33 |
| 38 | The place of KT in more global strategies within strategies for organizational change | 3.22 |
|  | **Average:** | **3.68** |
|  | **Cluster 9. Adaptation of KT strategies to context** |  |
| 11 | Relationships among the type of problem, the context, and KT | 4.11 |
| 36 | Issues and differentiated strategies with regards to tacit and explicit knowledge | 4.11 |
| 53 | The integration of existing evidence and knowledge within organizations | 3.89 |
| 45 | Variations in the operating mechanisms of KT based on the type of research and the knowledge produced | 3.89 |
| 48 | Variations in the operating mechanisms of KT based on the context in which it is used | 3.67 |
| 33 | The optimal relationship between tacit and explicit knowledge | 3.33 |
| 46 | Comparative analysis of health practices with other key sectors of society | 2.89 |
| 91 | Contamination strategies versus dissemination strategies in the adoption and use of knowledge | 2.78 |
|  | **Average:** | **3.58** |
|  | **Cluster 10. Theoretical research on KT** |  |
| 50 | Definition and measurement of knowledge transfer | 4.44 |
| 80 | State of knowledge regarding KT | 4.11 |
| 86 | The development of relevant methodological approaches for KT (specifications...) | 3.89 |
| 85 | The modalities for transposing research findings into models for action | 3.89 |
| 75 | The potential of knowledge syntheses for driving KT | 3.89 |
| 68 | The attributes of research-based knowledge that make it shareable and usable | 3.78 |
| 44 | Elaboration of a more in-depth theory on the use of knowledge | 3.67 |
| 78 | The definition of knowledge (object versus representation) | 3.67 |
| 74 | Factors contributing to the increase of KT | 3.56 |
| 16 | Specificity of KT based on field of application (social, health...) | 3.44 |
| 103 | Establishing a consensus on the meaning of terms in KT | 3.33 |
| 88 | The ‘science’ of recommendation | 3.22 |
| 102 | The French fact in the field of KT | 3.22 |
| 47 | An understanding of the determinants of KT depending on whether management or intervention is targeted | 3.11 |
| 104 | Abandoning obsolete knowledge in organizations | 3.11 |
| 59 | Historical perspective on the importance of KT in the health sector | 2.89 |
| 87 | Guidelines on how to interpret research findings in order to translate them into action when there is insufficient knowledge | 2.78 |
| 40 | Identifying opponents to KT | 2.78 |
| 22 | Modelling the impact of the organization’s KT on clients | 2.67 |
| 56 | Empirical markers of evidence-informed organizational change | 2.67 |
| 92 | The difference between the role of research in changing ideas versus its role in influencing specific decisions | 2.67 |
| 77 | The various definitions of client in the transformation of practice | 2.56 |
| 29 | Preparation of practical KT based on evidence about KT | 2.44 |
| 62 | The impact of KT on criteria for scientificity | 2.33 |
|  | Average: | 3.25 |

# Tables

## Table S2 - Statements by cluster for the health and social services group

|  | **Cluster 1. Power and organizational culture** |  |
| --- | --- | --- |
| 17 | Analysis of power (decision-making, influence, and recommendation) issues between researchers and caregivers | 3.70 |
| 71 | The use of research findings in relationships of power within organizations | 3.50 |
| 58 | Systemic issues surrounding the adoption and use of knowledge | 3.40 |
| 1 | The study of power sharing in decision making and action taking | 3.30 |
| 104 | Abandoning obsolete knowledge in organizations | 3.20 |
| 26 | Conditions for bringing professional cultures together in the context of establishing networks | 2.80 |
| 13 | Monitoring processes for decision-making elements | 2.30 |
|  | **Average:** | **3.17** |
|  | **Cluster 2. Conditions for implementing KT in organizational processes** |  |
| 79 | Organizational conditions for implementing KT activities | 4.00 |
| 61 | The strategic use by decision makers of research-based knowledge | 3.90 |
| 38 | The place of KT in more global strategies within strategies for organizational change | 3.90 |
| 99 | Strategies that promote continuity in the use of knowledge | 3.90 |
| 8 | The distinctive place of knowledge in decision-making processes through various organizational factors | 3.80 |
| 47 | An understanding of the determinants of KT depending on whether management or intervention is targeted | 3.80 |
| 18 | Institutional barriers and facilitating factors experienced by researchers and users | 3.60 |
| 68 | The attributes of research-based knowledge that make it shareable and usable | 3.60 |
| 2 | Preliminary capacities to build within systems (generation, dissemination, absorption) | 3.60 |
| 53 | The integration of existing evidence and knowledge within organizations | 3.50 |
| 74 | Factors contributing to the increase of KT | 3.50 |
| 12 | Capacity of KT mechanisms to adjust to the decision-making window | 3.40 |
| 10 | Receptivity of caregivers to new knowledge that will transform practice | 3.40 |
| 19 | Analysis of the degree of adoption of proven practices and their determinants | 3.30 |
| 51 | Development of a KT culture in order to reduce resistance to change | 3.10 |
| 60 | The concordance between the values stated by institutions and their KT practices | 2.90 |
| 42 | An understanding the processes of change in new public health practices | 2.90 |
| 93 | The availability of explicit scientific knowledge in organizations | 2.80 |
|  | **Average:** | **3.49** |
|  | **Cluster 3. Strategies adapted to the user’s needs** |  |
| 34 | The relevance of various KT strategies in relation to the needs of different types of users | 4.20 |
| 23 | Differentiated evaluation of the knowledge needs of the various stakeholders within the network | 3.60 |
| 72 | The role of patient populations and communities in KT strategies | 3.40 |
| 27 | Anticipation of the needs of users so that research findings are available when needed | 3.10 |
| 91 | Contamination strategies versus dissemination strategies in the adoption and use of knowledge | 2.80 |
| 32 | The ability to listen to the real and current needs of practitioners and researchers | 2.70 |
| 77 | The various definitions of client in the transformation of practice | 2.40 |
| 59 | Historical perspective on the importance of KT in the health sector | 1.90 |
|  | **Average:** | **3.01** |
|  | **Cluster 4. Contextualization of KT to the organizational setting** |  |
| 16 | Specificity of KT based on field of application (social, health...) | 3.90 |
| 48 | Variations in the operating mechanisms of KT based on the context in which it is used | 3.80 |
| 85 | The modalities for transposing research findings into models for action | 3.80 |
| 87 | Guidelines on how to interpret research findings in order to translate them into action when there is insufficient knowledge | 3.40 |
| 43 | Elements of recognition of this new practice among researchers as well as among users and their organizations | 3.30 |
| 46 | Comparative analysis of health practices with other key sectors of society | 3.10 |
| 11 | Relationships among the type of problem, the context, and KT | 2.90 |
| 81 | The impact of cultural differences (societal cultures) on KT | 2.60 |
| 35 | The place of KT research in clinical informatics | 2.40 |
|  | **Average:** | **3.24** |
|  | **Cluster 5. Links between different forms of knowledge** |  |
| 101 | The fit between scientific knowledge and field-based knowledge | 3.90 |
| 33 | The optimal relationship between tacit and explicit knowledge | 3.80 |
| 67 | The fit between the portrayals of knowledge by practitioners and researchers | 3.50 |
| 36 | Issues and differentiated strategies with regards to tacit and explicit knowledge | 3.50 |
|  | **Average:** | **3.68** |
|  | **Cluster 6. Characteristics, roles and dynamics of the actors** |  |
| 52 | The active role of practitioners and decision-makers in KT strategies | 3.50 |
| 6 | Roles and competencies to develop in research settings | 3.50 |
| 5 | The role of users in KT | 3.50 |
| 7 | Roles and competencies to develop in practitioner settings | 3.50 |
| 31 | The impact on knowledge producers of participating in KT | 3.40 |
| 57 | Study of the values and attitudes of the heads of universities and funding organizations towards KT | 3.20 |
| 63 | The professional and relational qualities of KT professionals | 3.20 |
| 14 | The abilities of professionals to identify relevant evidence | 3.00 |
| 94 | The responsibility of researchers to share knowledge resulting from their own research | 2.90 |
| 20 | Identify the skills of researchers in translating their data for different settings | 2.80 |
| 54 | Skill development in researchers for producing research on KT | 2.50 |
| 39 | The skills to develop in researchers for communicating with journalists and the media | 2.10 |
|  | **Average:** | **3.09** |
|  | **Cluster 7. Optimal modalities for collaboration between researchers and practitioners** |  |
| 9 | Modalities of collaboration between researchers and practitioners that foster the development of new practices | 3.80 |
| 64 | The role of communities of practice in creating synergy between tacit and explicit knowledge | 3.80 |
| 41 | The development of leadership roles related to KT on the ground | 3.70 |
| 83 | The characteristics of optimality in interdisciplinary teams engaged in KT | 3.70 |
| 24 | The views of researchers and practitioners on the deliberations necessary for using knowledge | 3.60 |
| 4 | The question of relational contexts that foster the use of knowledge | 3.60 |
| 95 | The importance of a relationship of trust between researchers and practitioners in the use of research-based knowledge | 3.50 |
| 88 | The ‘science’ of recommendation | 3.50 |
| 49 | Optimal positioning of KT professionals within the network | 3.20 |
| 25 | Strategies and processes for networking and partnership that foster the co-generation of knowledge | 3.00 |
| 40 | Identifying opponents to KT | 2.50 |
|  | **Average:** | **3.45** |
|  | **Cluster 8. Evaluation of effects and impacts** |  |
| 45 | Variations in the operating mechanisms of KT based on the type of research and the knowledge produced | 4.10 |
| 98 | Measuring the outcomes and benefits of KT | 4.10 |
| 3 | Assessment of experiments underway in order to identify best practices | 4.10 |
| 76 | Economic impact of KT practice on health systems | 4.00 |
| 29 | Preparation of practical KT based on evidence about KT | 3.90 |
| 15 | Analysis of the degree of adoption of proven practices and their determinants | 3.70 |
| 103 | Establishing a consensus on the meaning of terms in KT | 3.40 |
| 75 | The potential of knowledge syntheses for driving KT | 3.20 |
| 37 | The role of various KT strategies in developing competencies in public health in organizations | 3.00 |
| 82 | Transfer guides which are not evidence-based | 2.80 |
| 28 | The influence of an increasing demand for KT on the development of scientific careers, the development of methods, and on scientific performance | 2.70 |
| 89 | The impact of KT on population health | 2.60 |
|  | **Average:** | **3.47** |
|  | **Cluster 9. Theories, concepts, methods and measures of KT** |  |
| 50 | Definition and measurement of knowledge transfer | 4.10 |
| 56 | Empirical markers of evidence-informed organizational change | 3.70 |
| 44 | Elaboration of a more in-depth theory on the use of knowledge | 3.70 |
| 86 | The development of relevant methodological approaches for KT (specifications...) | 3.40 |
| 84 | The optimal modalities of KT: the role of researcher versus that of KT professional | 3.40 |
| 78 | The definition of knowledge (object versus representation) | 3.30 |
| 22 | Modelling the impact of the organization’s KT on clients | 2.90 |
| 92 | The difference between the role of research in changing ideas versus its role in influencing specific decisions | 2.80 |
| 55 | The proportion of resources allocated to KT in relation to the proportion of resources allocated to knowledge production | 2.50 |
| 80 | State of knowledge regarding KT | 2.50 |
| 62 | The impact of KT on criteria for scientificity | 2.50 |
| 97 | The impact of new communication technologies on KT | 2.40 |
| 102 | The French fact in the field of KT | 1.80 |
|  | **Average:** | **3.00** |
|  | **Cluster 10. Ethical issues** |  |
| 73 | The ethical challenges related to KT | 3.60 |
| 96 | The power held by knowledge brokers and the ethical issues this raises | 3.40 |
| 90 | The ethics of the role of the KT professional within the decision-making process | 3.20 |
|  | **Average:** | **3.40** |
|  | **Cluster 11. KT in the public arena** |  |
| 30 | The place of population needs in supporting choices | 3.10 |
| 65 | The place of formal knowledge in the evolution and moulding of public opinion | 2.70 |
| 100 | The impact of new communication technologies on how the population expresses its needs | 2.70 |
| 70 | Analysis of the population’s reception of speeches given by KT leaders (media, health agencies, etc.) | 2.10 |
| 21 | Methodological skills of journalists in understanding science | 2.00 |
| 66 | Media discourse on KT research activities | 2.00 |
| 69 | Public opinion of research on KT | 1.60 |
|  | **Average:** | **2.31** |
